# Reduced Self-Control after 3 Months of Imprisonment; A Pilot Study

**DOI:** 10.3389/fpsyg.2018.00069

**Published:** 2018-02-01

**Authors:** Jesse Meijers, Joke M. Harte, Gerben Meynen, Pim Cuijpers, Erik J. A. Scherder

**Affiliations:** ^1^Section Clinical Neuropsychology, Department of Clinical, Neuro- and Developmental Psychology, Vrije Universiteit Amsterdam, Amsterdam, Netherlands; ^2^Department of Criminal Law and Criminology, Vrije Universiteit Amsterdam, Amsterdam, Netherlands; ^3^Department of Philosophy, Faculty of Humanities, Vrije Universiteit Amsterdam, Amsterdam, Netherlands; ^4^Tilburg Law School, Tilburg University, Tilburg, Netherlands; ^5^Section Clinical Psychology, Department of Clinical, Neuro- and Developmental Psychology, Vrije Universiteit Amsterdam, Amsterdam, Netherlands; ^6^EMGO Institute for Health and Care Research, Vrije Universiteit Amsterdam and VU Medical Center Amsterdam, Amsterdam, Netherlands

**Keywords:** prison, executive functions, impoverished environment, self-control, impulsivity, offenders, CANTAB

## Abstract

**Background:** Prison can be characterized as an impoverished environment encouraging a sedentary lifestyle with limited autonomy and social interaction, which may negatively affect self-control and executive function. Here, we aim to study the effects of imprisonment on self-control and executive functions, and we report the change in neuropsychological outcome after 3 months of imprisonment.

**Materials and Methods:** Participants were 37 male inmates in a remand prison in Amsterdam, Netherlands, who completed six tests of a computerized neuropsychological test battery (the Cambridge Automated Neuropsychological Test Battery) in the first week of arrival. Participants were retested after 3 months of imprisonment. Change in performance was tested using the Wilcoxon Signed-Rank test.

**Results:** After 3 months of imprisonment, risk taking significantly increased (measured as an increase in the proportion of available points used for betting) and attention significantly deteriorated (measured as increased variability in reaction times on a sustained attention task), with large to medium effect sizes. In contrast, planning significantly improved (measured with a task analog to the Tower of London) with a medium effect size.

**Discussion:** Our study suggests that 3 months of imprisonment in an impoverished environment may lead to reduced self-control, measured as increased risk taking and reduced attentional performance. This is a significant and societally relevant finding, as released prisoners may be less capable of living a lawful life than they were prior to their imprisonment, and may be more prone to impulsive risk-taking behavior. In other words, the impoverished environment may contribute to an enhanced risk of reoffending.

## Introduction

Currently, more than 11 million people are imprisoned worldwide, and this number continues to rise (Walmsley, 2016). Imprisonment is characterized by a sedentary lifestyle (Young et al., 2005; Ireland and Culpin, 2006; Cashin et al., 2008; Elger, 2009; Plugge et al., 2009). For example, many prisoners do not meet the generally accepted norm of 30 min of moderate physical activity per day, and one study in particular found that UK prisoners tend to sit or lie on their beds for more than 9 h per day (Ireland and Culpin, 2006). Imprisonment also inherently results in decreased autonomy – as many responsibilities and decisions are shifted to the prison staff (Woodall et al., 2013) – and in social isolation, as prisoners are largely isolated from their own social networks.

Due to the sedentary lifestyle, social isolation, and a lack of cognitive challenges, prison can be considered an impoverished environment, which may negatively affect executive functions and prefrontal functioning of the brain (Scherder et al., 2010; Meijers et al., 2015a). Executive functions are top-down cognitive functions crucial for self-control (Nigg, 2016), such as planning, attention, working memory, set-shifting, and inhibition, largely regulated by the prefrontal cortex of the brain (Diamond, 2013). The prefrontal cortex is also important for bottom-up self-control: by enhancing activity in subcortical areas that favor an appropriate response, subcortical areas favoring inappropriate or impulsive responses are subsequently (thus indirectly) inhibited (Nigg, 2016). Besides the potential negative influence of the impoverished environment on self-control and prefrontal functioning, indirect consequences of an impoverished environment, such as chronic stress and sleep disturbances, from which prisoners often suffer (Feron et al., 2005; Ireland and Culpin, 2006; Nesset et al., 2011; Meijers et al., 2015b), may also negatively influence prefrontal functioning. Sleep disturbances are also considered to be a risk factor for aggressive behavior, especially in a high-risk population (Kamphuis et al., 2012).

Reduced prefrontal structure and functioning, impaired executive functions, as well as impulsivity (the result of a failure in bottom-up self-control), are hallmark features of antisocial and criminal populations (Yang and Raine, 2009; Kavanagh et al., 2010; Ogilvie et al., 2011). Although prospective studies are lacking, some studies show a relationship between executive or prefrontal dysfunction and increased reoffending (Santos Barbosa and Coelho Monteiro, 2008; Hancock et al., 2010). The question arises whether imprisonment in an impoverished environment may lead to a decline – or a further decline – in prefrontal functioning and self-control, which, in turn, may lead to increased risk of criminal recidivism.

In the current study, we investigate whether imprisonment reduces executive functions and self-control. To our knowledge, this is the first prospective study to address this question. We focused on change in impulsive risk taking (bottom-up self-control) and executive function (top-down self-control) in remand prisoners after 3 months of imprisonment, and hypothesize an overall decline in executive functions and self-control.

## Materials and Methods

### Participants

Participants were recruited between 2013 and 2015 at the Penitentiary Institution Amsterdam Over-Amstel – a remand prison in Amsterdam, Netherlands. We tested 130 male remand prisoners in their first week of imprisonment (T1), a comprehensive description of this population has been given elsewhere (Meijers et al., 2017). After 3 months (T2), we retested 37 of the participants (mean age = 30.5, *SD* = 9.9). Characteristics of this retested sample can be found in the section “Results” (**Table 1**). We elaborate on our choice to retest participants after 3 months and on the dropout rate in the section “Results,” as well as in the limitations section of the “Discussion.”

**Table 1:** Study sample characteristics (*N* = 37).

Characteristic	*M* (*SD*) or No. (%)
Age	30.54 (9.9)
Education level^a^	4.73 (1.1)
Estimated IQ^b^	89.31 (18.8)
Number of previous detentions	3.86 (4.7)
Number of days spent imprisoned during previous detentions	334 (418)
Violent offenders	20/37 (54%)
Estimated hours per day spent in bed during daytime^c^	6.54 (4.2)


*^*a*^Education level according to Verhage, a commonly used scale in the Netherlands ranging from 1 to 7. ^*b*^Estimated using Block Design and Information of the WAIS-IV. ^*c*^Self-reported estimate of the participants at T2.*

### Materials

Six tests of the Cambridge Automated Neuropsychological Test Battery (CANTAB, Cambridge Cognition, Cambridge, United Kingdom) were used to assess executive functions. Interactive demos and extensive descriptions of these tests are available at manufacturer website^1^. The tests were administered on a Windows 7, 12.1″ by CANTAB recommended touchscreen tablet, with a screen resolution of 1280 ^∗^ 800. The reported test–retest correlations for the subtests are 0.6–0.9 (Lowe and Rabbitt, 1998; Barnett et al., 2016). The following six subtests were used and administered – in the order described below – for all participants, at baseline (T1) and after 3 months (T2).

Stockings of Cambridge (SOC), analog to the commonly used Tower of London, measures planning. Presented with a horizontally split screen, participants are instructed to copy the pattern of colored balls in the upper half, by moving the colored balls in the lower half. Difficulty increases from two to five moves that are minimally needed, using the “clinical – no follow” mode. The main outcome variable is the *number of problems solved in the minimum required moves*.

The Spatial Working Memory (SWM) task measures working memory. When presented with multiple closed colored square boxes, participants are instructed to search for a small blue square hidden within one of the closed boxes. The closed boxes will contain a blue square only once; participants therefore have to remember in which box they already found a blue square, and in which they did not. Using the shortened-3X3p-2X4-40-2X6-60-2X8-80 mode, there are six assessed trials, with two each of four, six, and eight boxes. Looking inside a closed box that already contained a blue square once is classified as a *between error*. Looking inside a closed square twice within the same search is classified as a *within error*. The main outcome variable is *total errors* (*adjusted*).

The Stop Signal Task (SST) is a classic stop signal response inhibition test that measures response inhibition. This task uses a two-button press pad instead of the touch screen. Participants are instructed to press the left or right button as fast as they can, when they are presented with an arrow pointing to the left or right. After an initial practice set, participants are instructed to withhold their response when they hear an auditory signal (a beep). Using the clinical mode, there are five assessed blocks, each of 64 trials. Each block is divided into 4 sub-blocks of 16 trials; every sub-block contains, randomly played, 12 “go” trials, without an auditory tone, and 4 “stop” trials, with an auditory tone played following the “stop signal delay” (SSD) period. The SSD adjusts to the performance of each individual participant in such a way that successful stopping occurs in approximately 50% of the “stop” trials. The main outcome variable is the *stop-signal reaction time* (*SSRT*), which is calculated by subtracting the *SSD* from the *mean reaction time* (*MRT*).

The Intra-Extra Dimensional (IED) Set-Shift task measures set-shifting. Participants are presented with two clearly distinct types of stimuli, i.e., purple colored shapes and white lines, and learn which stimulus they should choose through feedback. After six correct responses, an intra-dimensional shift occurs, i.e., the correct answer is switched from one purple shape to another purple shape. After a number of intra-dimensional shifts, extra-dimensional shifts start to occur, i.e., the correct answer switches from a purple colored shape to a white line. Using the clinical mode – or parallel mode 2 at T2 – the test consists of nine blocks, through which the participant progresses by giving six consecutive correct answers. The test is aborted if a participant has a total of 50 incorrect responses within one block. The main outcome variable is *total errors* (*adjusted*).

The Choice-Reaction Time (CRT) task measures sustained attention. Similar to the first stage of the SST, participants are instructed to press the left or right button as fast as possible, when the corresponding left/right pointing arrow is presented on the screen. Using the clinical mode, the tests consist of one practice block of 24 trials, and two assessments blocks of 50 trials each. The main outcome variables are *MRT*, and *SD reaction time*, indicating the variability in reaction time.

The Cambridge Gambling Task (CGT) measures impulsivity and risk-taking. Under 1 of 10 either red or blue squares that are presented on the top of the screen, a yellow square is hidden by the computer each round. Participants are instructed to make as much profit as possible, by repeatedly betting a proportion of their points on one of the two possible outcomes (red or blue). Using the ascending first – shortened mode, the test consists of five stages. The first stage is a decision-only stage (without betting), so the participant can learn the decision-making process. The second and fourth stages are training stages, where the participant learns that the stakes either ascend or descend. The third and fifth stages are the actual test stages, each consisting of two blocks with nine trials. The main outcome variables are *overall proportion bet* and *delay aversion*.

Intelligence was estimated using two subtests, Information and Block Design, of the Wechsler Adult Intelligence Scale – Fourth Edition (WAIS-IV). This short form highly correlates (*r* = 0.931) with full-scale IQ (Girard et al., 2015).

The most common DSM-IV axis I diagnoses, as well as antisocial personality disorder and addiction, were screened for using the MINI International Neuropsychiatric Interview 5.0.0 – a short, structured diagnostic interview (Sheehan et al., 1998; Van Vliet and De Beurs, 2007).

Self-reported general mental and physical symptoms, such as pain, depression, and hopelessness, were assessed using the Symptom-Checklist-90 (Derogatis, 1996). Test–retest correlations for the SCL-90 range from *r* = 0.68 to 0.80 (Derogatis and Savitz, 2000).

### Procedure

We collected basic demographic data and information, such as criminal history, from the prison’s administrative databases. On a weekly basis, newly detained eligible prisoners were approached. Suspects of more serious crimes (e.g., murder, arson, rape, or aggravated assault) were prioritized in order to account for their lower prevalence compared to less serious, non-violent crimes (e.g., shoplifting). Prisoners were excluded if their stay was of transient nature, e.g., when they were awaiting extradition, when they were scheduled to be transferred to a different facility or scheduled for deportation to their home country. In a few exceptional cases, the prison staff did not allow us to approach specific prisoners, due to safety concerns. Further exclusion criteria included active psychosis, insufficient understanding of the Dutch or English language, visual or motor impairments to such a degree that tests cannot be seen or executed properly, insufficient understanding of the goal of the study or conditions concerning participation, and aggressive or inappropriate behavior toward the researcher. All approached prisoners were verbally invited to consider participating in our study. They were informed about the study’s goal and conditions, the tests that were used and the estimated time it would consume. A more extensive information letter was handed out to those interested, which could be read after this short introduction. An appointment was scheduled for the test administration with the prisoners who were willing to participate. We emphasized their right to cancel that appointment and to withdraw from the study at any given time without any consequences. We estimate the recruitment success percentage to be around 60%.

The current paper is part of a larger study encompassing a number of measures that are not used in the current paper, i.e., heart rate, measured with the Vrije Universiteit-Ambulatory Monitoring System (VU-AMS), and physical activity, measured with the IPAQ and Actical. The following section describes the procedure for the measures used in the current paper.

Testing took place within 7 days after the participants’ arrival in the Penitentiary Institution. T1 consisted of two separate appointments. Each first appointment started with the opportunity for the participant to ask questions about the study and the information letter. Next, the informed consent form was explained, and signed by both the participant and the researcher. We collected data on medication history and current use, history with drug abuse, education level, history with traumatic brain injury, and other relevant medical history during anamnesis. We also inquired about recent drug use within the institution. After they were informed about the confidentiality, a small number of participants reported recent drug use, which led to rescheduling or cancelling of the appointment.

After anamnesis, we administered the CANTAB tests, with an average duration of an hour, and asked the participants to fill in the SCL-90 at their own convenience. The second T1-appointment was planned within 21 days of arrival at the Penitentiary Institution, rather than 7 days, as IQ and psychiatric diagnoses are relatively stable in nature and thus less sensitive to the hypothesized negative influence of the prison environment. At this second T1-appointment, we administered the two WAIS subtests and the MINI, and answered questions that participants may have had about the SCL-90. At the end of the second appointment, participants were informed that we would approach them after approximately 3 months – if they were still imprisoned at the Penitentiary Institution of Amsterdam Over-Amstel at that time – to inquire whether they were still prepared to be retested. After 3 months (T2), participants were retested according to the procedure of the first appointment at T1.

This study was carried out in accordance with the recommendations of Ethics Committee for Legal and Criminological Research of the Faculty of Law, Vrije Universiteit Amsterdam with written informed consent from all subjects. All subjects gave written informed consent in accordance with the Declaration of Helsinki. A statement of the accredited medical ethical committee of the Vrije Universiteit Medical Center was provided that the study requires no further ethical approval. This study has been registered in the Dutch Trial Register (NTR5443^2^). Prisoners did not receive an incentive for participation, as the Custodial Institutions Agency did not allow us to do so. However, spending more time outside the prison cell is often considered as an incentive in itself, regardless of the activity. The safety of the participants and the researchers was guaranteed by the Penitentiary Institution. Data were stored according to the regulations for scientific research of the Custodial Institutions Agency (Dutch acronym: DJI) and will be saved for 15 years.

### Statistical Analyses

SPSS version 23 was used to analyze the data. We used ANOVA to analyze whether the participants who were tested at T2 differed – at T1 – from the participants not tested at T2, on any of the neuropsychological outcome measures and other relevant variables, such as demographics and type of crime. Change in performance between T1 and T2 was tested using the robust, non-parametric Wilcoxon Signed-Rank test – because of a non-normal distribution for most of the variables and the presence of a number of outliers – with *P* < 0.00625 as the level of significance to correct for multiple testing (Bonferroni correction: *P* = 0.05/8 = 0.00625).

In G^∗^Power (Faul et al., 2007), with alpha = 0.05, power = 0.80, and effect size = 0.50, sample size calculation for the matched pairs Wilcoxon Signed-Rank test (T1-T2) resulted in *N* = 35.

## Results

As many of the participants who had been tested at baseline were released before reaching 3 months of imprisonment, a significant number of participants was not retested at T2. Besides a higher proportion of violent offenders, the group of participants that we retested at T2 did not differ from the group that we only tested at T1 on any of the neuropsychological outcome measures at baseline or other relevant demographic variables (*P* > 0.072). Characteristics of the retested participants can be found in **Table 1**.

To investigate the effect of imprisonment on EF, we analyzed change in performance (T1-T2) on six subtests of the neuropsychological test battery. Risk taking (CGT Overall Proportion Bet) significantly increased after 3 months of imprisonment with a large effect size, *N* = 29, *T* = 11, *P* < 0.001, *r* = 0.49. Attentional performance significantly decreased, reflected in a significant increase in CRT SD (variability in reaction time on a sustained attention task) at T2 with a medium effect size, *N* = 35, *T* = 16.95, *P* = 0.005, *r* = 0.33. In contrast, planning (SOC Problems solved in minimum moves) significantly improved with a medium effect size, *N* = 37, *T* = 11.71, *P* = 0.006, *r* = 0.34. No further significant changes were found, see **Table 2** for an overview of the outcome measures at T1 and T2.

**Table 2:** Results on the CANTAB outcome measures on T1 and T2.

	T1	T2			
					
CANTAB outcome measures	*M* (*SD*)	*M* (*SD*)	*N*	*P*-value	Effect size (*r*)
SOC problems solved in min. moves	7.81 (1.63)	8.92 (1.89)	37	**0.002**	**0.34**
SWM total errors	16.24 (9.91)	13.84 (11.99)	37	0.047	0.23
SST SSRT	190.66 (59.46)	171.08 (54.83)	36	0.157	0.17
IED stages completed	7.86 (1.87)	8.03 (1.92)	36	0.496	0.08
CRT mean correct latency	309.63 (51.39)	316.79 (64.02)	35	0.704	0.04
CRT SD correct latency	72.03 (38.29)	84.62 (48.32)	35	**0.005**	**0.33**
CGT delay aversion	0.291 (0.166)	0.235 (0.176)	29	0.132	0.20
CGT overall proportion bet	0.507 (0.151)	0.605 (0.133)	29	**<0.001**	**0.49**


*Bold: significant at *P* < 0.00625. Effect sizes – small: *r* = 0.1, medium: *r* = 0.3, large: *r* = 0.5 (Cohen, 1992). Abbreviations: SOC, Stockings of Cambridge (planning); SWM, Spatial Working Memory; SST SSRT, Stop Signal Task Stop Signal Reaction Time (response inhibition); IED, Intra-Extra Dimensional Set-Shift task (set-shifting); CRT, Choice-Reaction Time (attention); CGT, Cambridge Gambling Task (risk taking behavior/self-control).*

## Discussion

The purpose of this study was to investigate the influence of imprisonment on executive functions and self-control. In line with our hypothesis of a decline in executive functions during incarceration, our results show a significant deterioration in self-control and attention after 3 months of imprisonment. Additionally, we observed an increased performance on a planning-task, which is in contrast with our hypothesis. Yet, it is noteworthy that improvements on neuropsychological tasks are often caused by practice effects (Beglinger et al., 2005), and improvements due to practice effects on the planning task were indeed found in a study aiming to determine the test–retest correlations of several CANTAB subtests (Lowe and Rabbitt, 1998). A control group would be needed to determine whether the increased performance in our sample is over and beyond such practice effects. Therefore, though participants may show improvements in planning, we emphasize the decline found in self-control and attention. As these are opposite to the expected improvements due to aforementioned practice effects, the effect sizes of the reduced self-control and attention may even be underestimated without the use of a control group.

The decrease in self-control was reflected on the CGT, measuring bottom-up self-control, while performance on the SST, measuring top-down response inhibition, remained constant. This finding suggests that the prison environment may impair bottom-up self-control specifically, while top-down response inhibition remains unaffected. Impaired bottom-up self-control may lead to impulsive risk taking in the face of reward: due to lowered prefrontal activity, a potential reward may be overestimated, while the potential negative consequences are underestimated. Although we found reduced response inhibition in violent offenders compared to non-violent offenders in our previous study (Meijers et al., 2017), one could argue that reduced bottom-up self-control may also exacerbate the risk for aggressive or violent behavior in high-risk individuals, as self-regulation is a complex interplay between both top-down and bottom-up inhibition (Nigg, 2016). Besides a decrease in bottom inhibition, we also found reduced attentional performance, reflected in the increased variability on the sustained attention task (CRT SD). Attention is highly related to self-control (Steimke et al., 2016), as sustained attention to a higher-level goal is a crucial prerequisite to self-control, further substantiating our finding of reduced self-control in our sample.

Although remarkably little is known about the effects of imprisonment on brain function and executive functions, as well as on the influence of imprisonment on reoffending and its possible underlying mechanisms (Nagin et al., 2009), recent studies do suggest that harsher – thus more impoverished – prison conditions may increase the risk of reoffending (Chen and Shapiro, 2007; Drago et al., 2011). Within the prison environment, limited autonomy, provocations, and temptations result in a reduced demand on a person’s self-control, or functions regulated by the prefrontal cortex, when compared to life outside of the prison walls. However, a successful return to society requires autonomous goal-directed behavior and self-control, as released prisoners are expected to refrain from further criminal behavior, and instead attain housing and a legitimate income. In fact, the impoverished prison environment may negatively affect executive functions needed for a successful return to society.

Even though we tested 130 prisoners at baseline, only 37 remained available willing to be retested at T2. However, when conducting such a study in a remand prison, researchers should always take into consideration that many of their participants will remain imprisoned for a limited amount of time. In fact, in Netherlands, remand prisoners are detained for 103 days on average – which is why we chose to retest after 3 months – and 55% is released within 1 month of imprisonment. To avoid a negative influence of the prison environment prior to testing, we deliberately chose to conduct our study in a remand prison among newly detained inmates. Future studies could consider focusing on convicted offenders who have awaited their trials at home. This would most probably reduce dropout significantly – since a suitable moment for T2 could be determined for each participant individually – and would also avoid the potential negative influence of imprisonment prior to testing. The question arises, however, how large the sample size would become when focusing on this very specific subgroup of offenders. Either way, the current study was a relatively small and explorative study that should be replicated in a larger sample and include a non-imprisoned control group. Future researchers should bear in mind that collecting data in a prison setting is rather time-consuming compared to most other settings (Meynen, 2017). Another consideration is whether participants should be fully informed about the study goals before testing. Participants may have their own theories around residing in an impoverished environment and the potential influence on their cognitive abilities, although the increased performance on one of our tasks implies that this does not have to lead to reduced performance.

In sum, the decline in self-control which we observed in prisoners is a significant and societally relevant finding, possibly also regarding criminal recidivism. Released prisoners may be less capable than they were before imprisonment to live a lawful life outside of crime and may be less able to sustain focus on higher-level goals and more prone to impulsive risk-taking behavior. This finding may also partly explain why several studies report a relationship between harsher prison environments and higher reoffending rates (Chen and Shapiro, 2007; Nagin et al., 2009; Drago et al., 2011). Our results call for further research regarding the impact of the prison environment on brain function and self-control, as well as the influence of the prison environment on reoffending. Ultimately, this may lead to the recommendation that prisons be transformed into enriched environments to increase, or at least preserve, prisoners’ self-control.

## Author Contributions

JM, JH, GM, and ES developed the study concept and contributed to the study design. JM tested and collected data, performed the data analysis and interpretation under the supervision of the other authors, and drafted the manuscript, and all other authors provided critical revisions. All authors approved the final version of the manuscript for submission.

## Conflict of Interest Statement

The authors declare that the research was conducted in the absence of any commercial or financial relationships that could be construed as a potential conflict of interest.

## Footnotes

1 www.cambridgecognition.com

2 www.trialregister.nl
